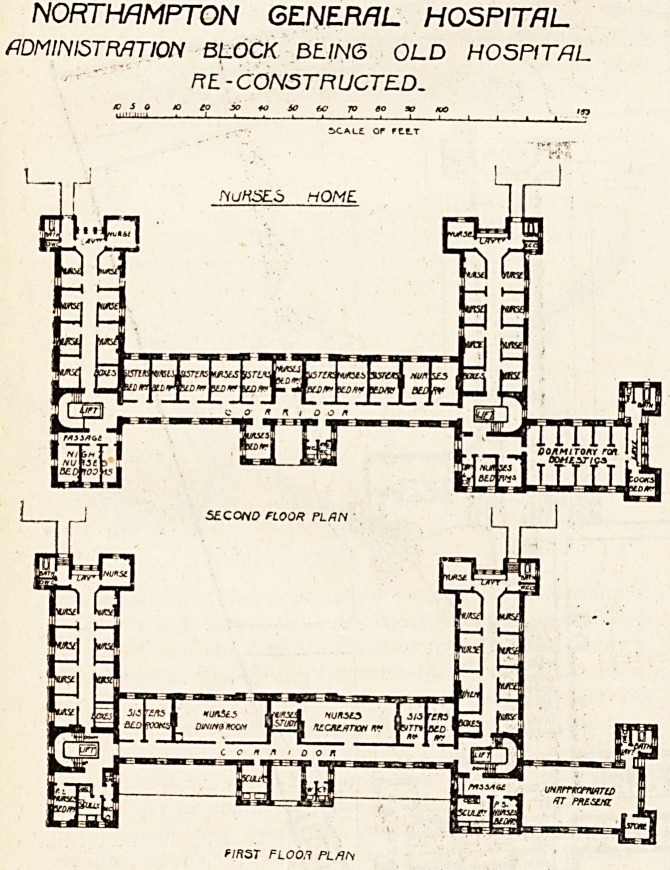# The Northampton Hospital

**Published:** 1907-09-14

**Authors:** 


### HOSPITAL ADMINISTRATION.

### CONSTRUCTION AND ECONOMICS.

### THE NORTHAMPTON HOSPITAL.

THIS hospital, known formerly as the Northampton General Infirmary, was a structure of various dates—a sort of conglomerate building—but by far the greater portion had been erected previous to the inauguration, or at least previous to the common adoption, of correct ideas of modern hospital architecture. Consequently, there had been a considerable amount of dissatisfaction among the honorary medical and surgical staffs regarding some parts of the building, and acting upon the advice of the staff, the managers requested Sir Henry Burdett to inspect the infirmary and report thereon. The greatest blemishes to the infirmary, as it then stood, were the surgical wards, the foul-air shafts, the sanitary annexes, and the poor accommodation for the nurses. It was thought that the two first named drawbacks might be remedied by rather extensive alterations in the wards themselves, the third by pulling down the annexes and rebuilding them with properly-constructed cross-ventilated lobbies, and the last by putting up an entirely new home for the nursing staff.

Subsequently, Mr. Frederick Dorman and Mr. Richard Greene were appointed architects, and a thorough examination was made by them of every part of the old structure.

NORTHAMPTON GENERAL HOSPITAL

GROUND FLOOR PLAN. 1905.

RICHARD GREEN F.R.C.P.E.

FRED. W. DORMAN A.R.I.B.A.

ARCHITECT.

ALL SAINTS CHAMBERS

NORTHAMPTON.

It was evident that, however economically the alterations were made, the expenditure of a very large sum of money would be required, and even then, it was pointed out by the architects, the hospital could not be a thoroughly satisfactory job. For instance, there was not a ward in which the windows were so arranged as to provide that every bed should have a window on both sides of it, which is one of the fundamental points in hospital construction. Further, the window heads were several feet below the ceiling levels. On examination, the architects were satisfied that the old walls could not safely be interfered with so as to permit the putting in of several new windows and altering the old ones, and moreover, there would have been great difficulty with the chimney breasts, which were awkwardly placed. However, two sets of plans were prepared. One on the lines as above suggested, and the other in which the old building was to be altered and almost entirely re-modelled so as to form a really good administrative department and a nurses' home, while the hospital proper was to be completely new, and to be connected with the old building by fire-proof bridges. These plans were fully described by Sir Henry Burdett at a general meeting of the Board, and it was decided to adopt the latter scheme, a decision which we think reflected great credit on the good judgment of the Board. The architects' estimate for this work was £30,000, and it was duly emphasised that the alternative scheme would have cost considerably more than half that sum, and that the result could never have been quite satisfactory, and could only have postponed for some years the day of rebuilding.

NORTHAMPTON GENERAL HOSPITAL
ADMINISTRATION BLOCK BEING OLD HOSPITAL RE-CONSTRUCTED.

We publish to-day the plans of the hospital as it now stands, and we shall begin our description by noticing the most important alterations made to the old building. The entrance-porch was not interfered with, but exactly south of this a small room was swept away giving place to a good lobby opening straight into the main corridor running north and south, and which in its turn joins the transverse corridor communicating with the new blocks.

On the east, and immediately on entering the old porch, is a suite of rooms for the secretary and his clerks, and beyond these rooms is the matron's store-room. On the west of the entrance are the porter's rooms, the waiting-room, and store-rooms. South of all these rooms is a passage, necessarily somewhat ill-lighted but improved in this respect, and south of this passage are the rooms for the resident medical staff, the dispensary, pantry, etc. Further east is the operating-theatre, with its adjuncts. This department being of recent construction was not interfered with, save that the arrangements for warming it were improved. From this wing a corridor leads to the out-patients' department, also not altered in any way, as it was comparatively modern, and near the corridor is a subway leading to the sub-ground level of the isolation block, the ground floor of which with its separate entrance on the ground level, contains the committee-rooms, and the first floor has been arranged and altered into isolation wards, approached by the sub-way already mentioned.

Returning to the old staircase, east wing, we enter the former male surgical ward. A passage has been cut off the east side of this ward, which passage leads direct to the new fire-proof bridge, hence there is direct access from the new surgical wards to the operating-theatre. The remaining part of the old ward has been divided into several rooms for the Röntgen Rays apparatus, the medical electrical instruments, the pathological laboratory, surgical appliances, etc. The corresponding wing on the women's side now contains the libraries and the matron's rooms. Close to the latter is the fire-proof bridge leading to the new west wing for female patients, while the wing corresponding in position to the operating theatres has been converted into a residence for the secretary.

As the new pavilions are identical it will be sufficient to describe one. Both are three stories high. Taking the east, or men's wing, and beginning at the south end of the fire-proof bridge on the ground-floor, we have on the left the nurses' lavatory and closet, and on the right the staircase and the electric lift. Crossing the transverse, or east and west corridor, we have before us on entering the wing the whole of the ward arrangements, or "hospital unit," as it is now called. On the left is a well-lighted room intended for minor operations, for the examination of patients, or for such general purposes as the honorary staff may think most useful for their several departments; and this room is fitted up with hot and cold douches, etc. On the right hand are the rooms for coal, linen, patients' clothing, brooms and pails, and beyond these is a large receptacle for soiled linen, so arranged that soiled articles can be at once removed to the laundry without anyone coming into the ward. South of all these rooms is a passage which on the left leads to a four-bedded ward having its own bath-room and closet. This ward is intended for special cases of any kind, or for private patients. On the right is the ward kitchen with a small scullery or pantry attached to it, and having a hatch for delivery of food straight to the ward. At the west end of the passage is a single-bedded ward. This room has two windows on its south wall and one on its north wall, and there is a large hopper fanlight over the door. It therefore possesses complete cross-ventilation, and it may be said that it is one of the very few single-bedded wards in any hospital for which so much can be said. Just before entering the main ward is seen the sisters' duty-room—a large, cheerful room with a window facing the south-east, and there is in it also an observation-window for overlooking the ward. The ward is 110 feet long and 24 feet wide, and it contains 20 beds.

Every bed has a window on both sides, and a wall space of 11 feet, a floor space of 132 square feet, which, with a 12 feet ceiling, would give nearly 1,600 cubic feet of air space per patient, an amount which is, perhaps, not equalled by any general hospital in England. The sanitary annexes project from the south end of the ward, and they are quite effectually cut off from the main building by cross-ventilated passages. Between these annexes is a large balcony, and from the balcony springs a fire-escape staircase. Here, it may be stated, that there are fire-mains on each floor, and that each ward has its own fire-proof bridge at one end, as well as the common fire-escape staircase at the other end. Every ward has its own lock-up poison cupboard flush with the wall, and the surgical wards contain cupboards under the sills for the preparation and storage of aseptic dressings. Such is the ground-floor "ward unit"; and the first and second floors have the same arrangements.

Of the old building, the first floor has been altered so that the former wards facing due south are now the nurses' dining-room and nurses' recreation-room, both of these being large and handsome apartments, and this front has also four bedrooms for nurses. The wing running north and south on the east side is now divided by a central corridor from which open nine rooms for nurses. At the south end of the corridor are the bath-room and closet, and near this point is the fire-proof bridge leading to the first floor of the new pavilion. The west wing has been similarly altered. The floor over the secretary's residence has been kept in its original state as an emergency ward for any such purpose as the treatment of an epidemic among the staff, or as a room which could be temporarily used for patients when any of the new wards required being "done-up." Besides, this ward was freer from objectionable features than any of the others, hence its retention.

The second floor has been subjected to alterations similar to the first floor, and it now contains rooms for 33 nurses. There are also rooms or cubicles for 13 domestics, and the latter have their own bath-room.

As regards construction of the new pavilions, it may be mentioned that the outer walls are exceptionally thick and are also buttressed, for greater security. The floors of the surgical wards and the corridors and annexes are laid down with terrazzo, and the rest of the ward floors and the doors are of teak. The windows are on the same principle as those of Guy's Hospital. The insides of the walls and partitions are covered with adamantine or some similar plaster, and are painted with ripolin. The bath-rooms, lavatories, and closets are fitted up with Shanks and Co.'s appliances. There are electric-fans in the wards, and the lighting is by electricity. The large wards have each four open fire-places, and these have been ingeniously arranged without having projecting chimney-breasts, and without interfering with the wall space for the beds. Hot water radiators are also used, and the radiators are on the hinge principle, so that they can be drawn out and the space kept clean. All angles of floors and ceilings have been rounded off. The total cost was £30,000, which sum not only included all the new work, all the extensive alterations to the old building, but also new furniture for the wards and for the nurses' rooms. The total accommodation is for 168 beds, and, therefore, the cost works out at about £180 per bed, a sum which cannot be regarded otherwise than as extremely low, considering the high class of work obtained. Indeed, it is doubtful whether any modern hospital has been built within 50 per cent. in excess of the sum here expended, even after making all due allowance for the value of the old structure. We think, in reviewing all the circumstances, that the architects, by their careful planning and specification, were instrumental in saving the managers about £15,000; while the managers themselves are to be congratulated on the bold manner in which they handled a rather difficult problem, and on the result of their labours, for they may feel sure that they now possess a main building far ahead in point of efficiency of the majority of provincial hospitals. There are, however, three adjuncts of which we hope the funds of the institution will ere long permit the erection. These are a new laundry, a mortuary and post-mortem room, and a new entrance lodge, the latter placed at some convenient point in Cheyne Walk.

The contractor for the work was Mr. Henry Martin, of Northampton. We have already given the architects' names.

## Figures and Tables

**Figure f1:**
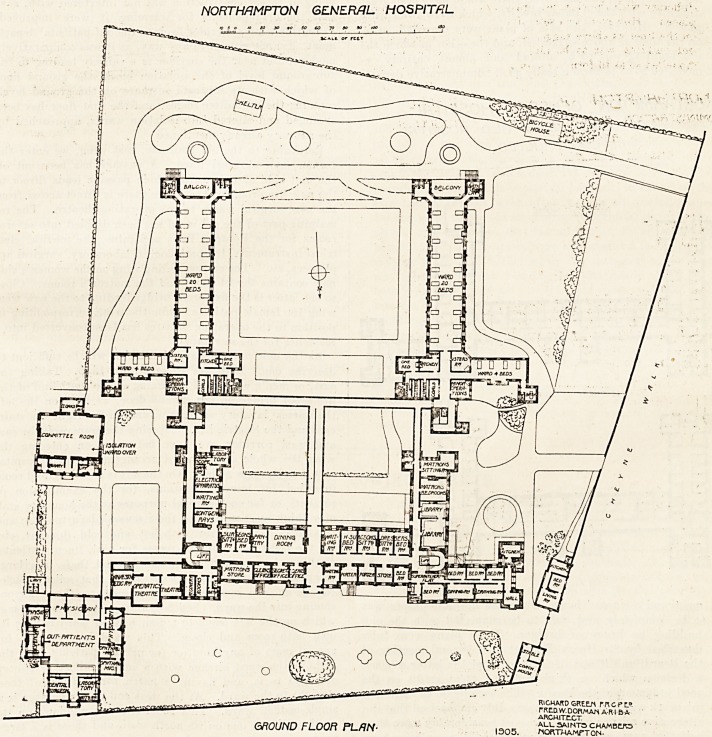


**Figure f2:**